# Sex-specific adverse event profiles of PDE4 inhibitors: a comparative big-data pharmacovigilance study of apremilast, crisaborole, and roflumilast in FAERS (2004–2025)

**DOI:** 10.3389/fphar.2026.1761303

**Published:** 2026-03-31

**Authors:** Baiqing Huang, Shaorui Gu, Siqi Wang, Yirou Ma, Yongxin Zhou, Wenli Wang

**Affiliations:** 1 Department of Cardiothoracic Surgery, Tongji Hospital, School of Medicine, Tongji University, Shanghai, China; 2 Department of Pharmacology, School of Medicine, Tongji University, Shanghai, China

**Keywords:** apremilast, crisaborole, disproportionality analysis, FAERS, pharmacovigilance, roflumilast, sex differences, time-to-onset

## Abstract

**Introduction:**

Phosphodiesterase-4 (PDE4) inhibitors, including apremilast, crisaborole and roflumilast, are widely prescribed for chronic inflammatory diseases. However, sex-specific safety profiles of these agents in routine clinical practice remain poorly characterized.

**Methods:**

We conducted a retrospective pharmacovigilance study using the US Food and Drug Administration Adverse Event Reporting System (FAERS) from Q1-2004 to Q1-2025. Data cleaning and deduplication were performed following the FDA Guidance for Industry: Pharmacovigilance Practices and Pharmacoepidemiologic Assessment (FDA, 2005). Specifically, deduplication was executed by retaining only the most recent FDA_DT for each unique CASEID, and demographic (DEMO), drug (DRUG), and reaction (REAC) files were merged. Reports listing apremilast, crisaborole, or roflumilast as primary suspect drugs formed three target cohorts. Disproportionality analyses were performed using reporting odds ratio (ROR), proportional reporting ratio (PRR), Bayesian confidence propagation neural network (BCPNN, information component), and multi-item gamma Poisson shrinker (MGPS, empirical Bayes geometric mean). Positive signals required meeting all four algorithmic thresholds. Signals were summarised at Preferred Term (PT) and System Organ Class (SOC) levels, stratified by sex, and visualised with volcano plots, forest plots, and SOC-level heatmaps. Time-to-onset (TTO) was calculated as the interval between treatment start and event onset.

**Results:**

A total of 127,516 Apremilast, 7,562 Crisaborole, and 2,142 Roflumilast reports were included. Clear sex-specific disproportionality patterns emerged across the three agents. For Apremilast, females exhibited higher reporting of dizziness, palpitations, and infection-related adverse events, whereas Crisaborole showed a modest male predominance for “product use issue,” and Roflumilast demonstrated male-skewed signals, particularly for malignancy- and metabolism-related events. System Organ Class (SOC) analyses further revealed distinct organ-system involvement for each agent. Time-to-onset profiles showed substantially delayed onset for Apremilast compared with the more immediate onset observed for Crisaborole and Roflumilast. The median TTO (IQR) was 24 (7–86) days for Apremilast, 4 (1–15) days for Crisaborole, and 42 (14–128) days for Roflumilast.

**Discussion/Conclusion:**

This large-scale real-world analysis reveals pronounced sex-specific and drug-specific heterogeneity in the safety profiles of PDE4 inhibitors. These findings highlight the need for sex-stratified risk communication, individualized monitoring strategies, and further mechanistic investigations.

## 1 Introduction

Phosphodiesterase-4 (PDE4) inhibitors constitute an important therapeutic class targeting cyclic adenosine monophosphate (cAMP) regulation and downstream inflammatory pathways. Currently approved agents—including Apremilast (oral), Crisaborole (topical), and Roflumilast (oral or topical formulations)—are widely used for psoriasis, psoriatic arthritis, atopic dermatitis, and chronic obstructive pulmonary disease (COPD), among other inflammatory and immune-mediated conditions (Kumar et al., 2013; Li et al., 2018). Although randomized clinical trials have established favorable efficacy and acceptable short-term tolerability profiles for these drugs, accumulating post-marketing evidence suggests a broader spectrum of adverse events (AEs) than previously recognized, with variations across organ systems and specific patient populations (Crocetti et al., 2022; Fan et al., 2024). Given the expanding real-world utilization of PDE4 inhibitors globally, a systematic characterization of their comparative safety profiles is urgently needed.

Drug safety assessment in clinical trials is inherently limited by restricted sample sizes, short follow-up periods, selected populations, and exclusion of patients with comorbidities or polypharmacy (Li et al., 2023; Linger and Martin, 2018). By contrast, the U.S. Food and Drug Administration (FDA) Adverse Event Reporting System (FAERS) offers a valuable resource for detecting rare, unexpected, or long-term AEs at a population scale (Li et al., 2025). FAERS-based disproportionality analyses have been widely applied to identify pharmacovigilance signals across dermatologic, immunologic, and respiratory therapeutics (Lei et al., 2025; Zhao et al., 2024; Que et al., 2025). However, despite the growing number of studies evaluating individual PDE4 inhibitors, no comprehensive class-level, multi-algorithm, real-world comparison of Apremilast, Crisaborole, and Roflumilast has been conducted. Likewise, existing literature has not examined organ-system patterns (SOC-level), onset timing, or cross-drug heterogeneity in a unified framework.

In addition to class-level safety, sex-specific differences in drug response and toxicity have increasingly attracted attention. Biological and immunological differences between males and females-including hormone-regulated signaling, body composition, metabolism, microbiome diversity, and immune responsiveness-are known to affect drug pharmacokinetics and pharmacodynamics (Zucker and Prendergast, 2020) (Dalla and Kokras, 2025). Regulatory agencies, including the FDA and NIH, have called for systematic integration of sex as a biological variable in safety research and risk communication (Evaluation of Sex Differences in, 2013) (NIH Policy on Sex as, 2015). Nevertheless, sex-stratified safety evidence for PDE4 inhibitors remains extremely limited. Only sporadic post-marketing observations have suggested that females may report more gastrointestinal or neuropsychiatric symptoms with Apremilast, whereas males may have distinct AE patterns with Roflumilast—yet these findings lack robust validation from large-scale real-world datasets (Shan et al., 2023). Moreover, no prior study has simultaneously evaluated sex-differential disproportionality, volcano-plot visualization, forest-plot quantification, and class-wide comparison across all three PDE4 inhibitors.

Given these major evidence gaps, a systematic investigation integrating real-world pharmacovigilance, multi-dimensional signal analytics, and sex-specific risk assessment is critically needed. In this study, we leveraged the FAERS database (Yan et al., 2025), comprising over 19million deduplicated reports, to perform a comprehensive evaluation of Apremilast, Crisaborole, and Roflumilast. Using four complementary disproportionality algorithms—reporting odds ratio (ROR), proportional reporting ratio (PRR), Bayesian confidence propagation neural network (BCPNN), and multi-item Gamma–Poisson Shrinker (MGPS) (Bate, 2007)—we profiled drug-specific and sex-specific AE signals across preferred term (PT) and system organ class (SOC) levels (Kong et al., 2025). Additionally, we examined time-to-onset (TTO) differences to explore early vs. delayed AE patterns among the three agents, in line with growing interest in temporal pharmacovigilance analyses (Liu et al., 2024; Zheng et al., 2025).

This work provides the first class-wide, sex-stratified, multi-algorithm pharmacovigilance analysis of PDE4 inhibitors in real-world practice, uncovering novel risk patterns and heterogeneity that may inform patient counseling, individualized monitoring, and safer prescribing strategies.

## 2 Methods

### 2.1 Data source and study design

This retrospective pharmacovigilance study used publicly available data from the FAERS. All quarterly files from Q1 2004 to Q1 2025 were downloaded, including the DEMO, DRUG, REAC, and THER (Therapy) datasets to ensure complete information for time-to-onset calculation. The FDA Adverse Event Reporting System (FAERS) consists of large-scale, spontaneously reported Individual Case Safety Reports (ICSRs). While the database follows a structured format—including demographic, drug, and reaction fields—the clinical narratives and reporting behaviors are inherently heterogeneous. Reports are coded using the Medical Dictionary for Regulatory Activities (MedDRA). The study followed FDA recommendations for FAERS data processing and reporting, and adhered to established pharmacoepidemiology practices for disproportionality analysis. As FAERS contains anonymized public data, institutional review board approval was not required.

### 2.2 Deduplication of FAERS reports

Deduplication was performed according to FDA recommendations to ensure that each case was counted once. Reports were grouped by CASEID, and for each group, the entry with the most recent FDA_DT and highest PRIMARYID was retained. Demographic inconsistencies and biologically implausible values were excluded. After deduplication, 19,026,509 unique ICSRs were included for analysis.

### 2.3 Adverse event processing

AEs were mapped to MedDRA PTs and SOC categories. Reports lacking AE information were excluded; those missing demographic fields were retained for overall analyses but excluded from sex-stratified evaluations.

### 2.4 Disproportionality analysis

A case–non-case framework was applied using two-by-two contingency tables (Table 1). Four major signal-detection algorithms were used: ROR, PRR, BCPNN, and MGPS. The formulas, output metrics, and positive-signal criteria for each algorithm are summarized in Table 2. A signal was deemed positive when all thresholds were met: ROR lower 95% CI > 1; PRR ≥2 with χ^2^ ≥ 4; IC025 > 0; and EBGM05 > 1.

**TABLE 1:** Two-by-two contingency table for disproportionality analyses. Abbreviations: AE (adverse event).

Group	Target AEs	All other AEs	Total
Target drug	a	b	a + b
All other drugs	c	d	c + d
Total	a + c	b + d	a + b + c + d

**TABLE 2:** Summary of major algorithms used for signal detection.

Algorithms	Indicator	Equation	Criteria
ROR	ROR	ROR = (ad)/(bc)	ROR05 > 1, N ≥ 2
​	​	95%CI = e^ln(ROR)±1.96(1/a+1/b+1/c+1/d)0.5^	​
PRR	PRR	PRR = [a/(a+b)]/[c/(c + d)]	PRR≥2
​	χ^2^	χ^2^ = [(ad-bc)^2^ (a+b + c + d)]/[(a+b)(c + d)(a+c)(b + d)]	χ^2^ ≥ 4, N ≥ 3
BCPNN	IC	IC = log_2_[a (a+b + c + d)]/[(a+c)(a+b)]	IC025 > 0
​	​	95%CI = e^ln(IC)±1.96(1/a+1/b+1/c+1/d)0.5^	​
MGPS	EBGM	EBGM = a (a+b + c + d)/(a+c)/(a+b)	EBGM05 > 1, N ≥ 3
​	​	95%CI = e^ln(EBGM)±1.96(1/a+1/b+1/c+1/d)0.5^	​

Abbreviations: ROR (reporting odds ratio); PRR (proportional reporting ratio); BCPNN (Bayesian confidence propagation neural network); MGPS (multi-item gamma Poisson shrinker); CI (Confidence Interval); IC025 (lower 95% credibility limit of Information Component); EBGM05 (Empirical Bayes Geometric Mean, lower 5% confidence limit).

### 2.5 Sex-specific disproportionality

Sex-stratified disproportionality was calculated by estimating ROR separately for males and females. Sex-differential risk was evaluated using log_2_(ROR_male/ROR_female), where positive values reflect male-predominant risk and negative values reflect female-predominant risk. Volcano plots were generated using log_2_ ratios and FDR-adjusted p-values. To quantify the magnitude of sex differences, the Ratio of Reporting Odds Ratios ( $ROR_{ratio}$ ) was calculated: $ROR_{ratio}=\frac{ROR_{male}}{ROR_{female}}$ Statistical significance for the $ROR_{ratio}$ was determined using a Z-test on the log-transformed RORs, followed by False Discovery Rate (FDR) correction via the Benjamini–Hochberg method.

### 2.6 System-level AE patterns

To evaluate cross-drug organ-system heterogeneity, significant PT-level signals were aggregated to SOC categories, and SOC-level RORs were calculated by identifying unique caseIDs within each SOC category. This case-level aggregation ensures that each individual report is counted only once per SOC, even if multiple PTs were reported, thereby avoiding the bias of double-counting. Heatmaps were constructed to visualize SOC-level patterns for each PDE4 inhibitor.

### 2.7 Time-to-onset analysis

Time-to-onset (TTO) was calculated as the interval between treatment start (START_DT, extracted from the THER or DRUG files) and adverse event onset (EVENT_DT). Data cleaning was performed to exclude reports where: (i) EVENT_DT occurred before START_DT; (ii) dates were incomplete (missing month or day); or (iii) TTO exceeded 20 years, which was considered biologically implausible.

### 2.8 Statistical software

All analyses were performed using R (version 4.4.2) with openEBGM and related packages. False discovery rate (FDR) correction used the Benjamini–Hochberg method, with significance defined as p < 0.05.

## 3 Results

**FIGURE 1:**
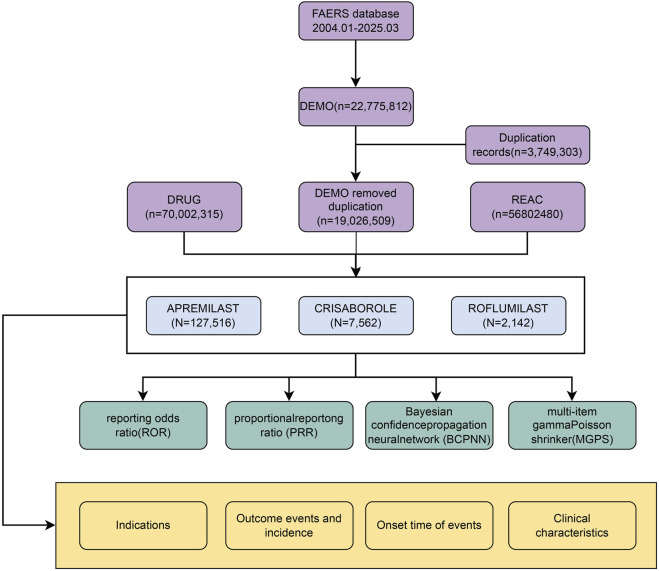
Overall study workflow for FAERS-based pharmacovigilance analysis. This flowchart presents the full data-processing pipeline, including FAERS data extraction (January 2004–March 2025), deduplication of DEMO records, extraction of DRUG and REAC files, identification of Apremilast, Crisaborole, and Roflumilast as primary suspect drugs, and subsequent analytical procedures. Four complementary algorithms—reporting odds ratio (ROR), proportional reporting ratio (PRR), Bayesian confidence propagation neural network (BCPNN), and multi-item Gamma–Poisson Shrinker (MGPS)—were applied for signal detection, followed by analyses of indications, adverse event profiles, onset time, and patient characteristics.

Across the deduplicated FAERS dataset (2004–2025Q1), a total of 137,220 adverse event (AE) reports were included for Apremilast (N = 127,516), Crisaborole (N = 7,562), and Roflumilast (N = 2,142). Baseline demographic, temporal, and indication distributions displayed substantial heterogeneity across the three agents, reflecting their distinct routes of administration, approved populations, and real-world utilization patterns (Table 3). The overall data extraction and analytic workflow is summarized in Figure 1.

**TABLE 3:** Baseline characteristics of FAERS reports for Apremilast, Crisaborole, and Roflumilast (2004–2025Q1).

Characteristics	Apremilast	Crisaborole	Roflumilast
​	(N = 127,516)	(N = 7,562)	(N = 2,142)
Gender			
Female	79,881 (62.6%)	4,333 (57.3%)	792 (37.0%)
Male	43,084 (33.8%)	2,270 (30.0%)	935 (43.7%)
Missing	4,551 (3.6%)	959 (12.7%)	415 (19.4%)
Age			
<18	327 (0.3%)	1907 (25.2%)	6 (0.3%)
18∼64	51,881 (40.7%)	2,526 (33.4%)	287 (13.4%)
65∼85	15,316 (12.0%)	1,078 (14.3%)	798 (37.3%)
>85	444 (0.3%)	67 (0.9%)	38 (1.8%)
Missing	59,548 (46.7%)	1984 (26.2%)	1,013 (47.3%)
Year			
2012	-	-	115 (5.4%)
2013	-	-	208 (9.7%)
2014	1,182 (0.9%)	-	226 (10.6%)
2015	11,102 (8.7%)	-	158 (7.4%)
2016	12,781 (10.0%)	-	156 (7.3%)
2017	18,403 (14.4%)	1,210 (16.0%)	120 (5.6%)
2018	19,422 (15.2%)	1841 (24.3%)	92 (4.3%)
2019	21,626 (17.0%)	1895 (25.1%)	158 (7.4%)
2020	18,089 (14.2%)	874 (11.6%)	89 (4.2%)
2021	9,812 (7.7%)	243 (3.2%)	136 (6.3%)
2022	5,965 (4.7%)	610 (8.1%)	131 (6.1%)
2023	4,728 (3.7%)	443 (5.9%)	288 (13.4%)
2024	3,537 (2.8%)	359 (4.7%)	210 (9.8%)
2025Q1	869 (0.7%)	87 (1.2%)	54 (2.5%)
Indication			
Psoriasis	74,808 (58.6%)	126 (1.6%)	108 (5.0%)
Psoriatic arthropathy	32,628 (25.5%)	5 (0.1%)	2 (0.1%)
Rheumatoid arthritis	1,501 (1.1%)	1 (0.1%)	7 (0.3%)
Hypertension	1,286 (1.0%)	18 (0.2%)	54 (2.5%)
Behcet'S syndrome	700 (0.5%)	-	-

This table summarizes demographic characteristics (sex and age), reporting year distribution, and primary indications of Apremilast (N = 127,516), Crisaborole (N = 7,562), and Roflumilast (N = 2,142) cases identified as primary suspect drugs in the FAERS, database. Missing values reflect unreported fields in spontaneous submissions.

### 3.1 Overall AE distributions, sex composition, and baseline characteristics

Apremilast users demonstrated the broadest demographic spread, with a majority of female reporters (62.6%) and a long usage history dating back to 2014. Crisaborole reports were predominantly pediatric and adolescent, consistent with its topical dermatologic indications, while Roflumilast users were mainly older males, consistent with its COPD indication. These characteristic differences provide clinical context for downstream AE divergence.

### 3.2 Sex-differential AE patterns and drug-level contrasts

**FIGURE 2:**
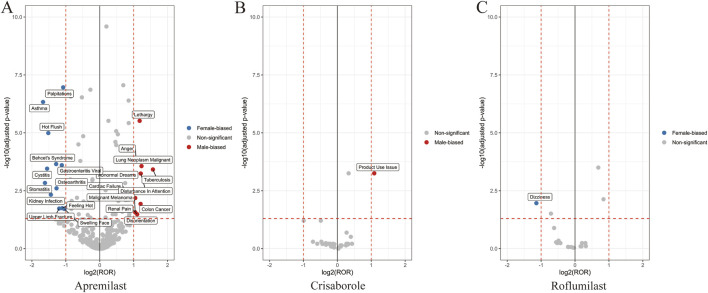
Sex-stratified volcano plots of adverse events for the three PDE4 inhibitors. **(A)** Apremilast. **(B)** Crisaborole. **(C)** Roflumilast. Each volcano plot displays log2 male-to-female reporting odds ratios (x-axis) against −log10 adjusted p-values (y-axis). Vertical dashed lines denote sex-biased thresholds; horizontal dashed lines indicate statistical significance. Blue or red points indicate significant sex-biased preferred terms; gray points indicate non-significant signals.

**FIGURE 3:**
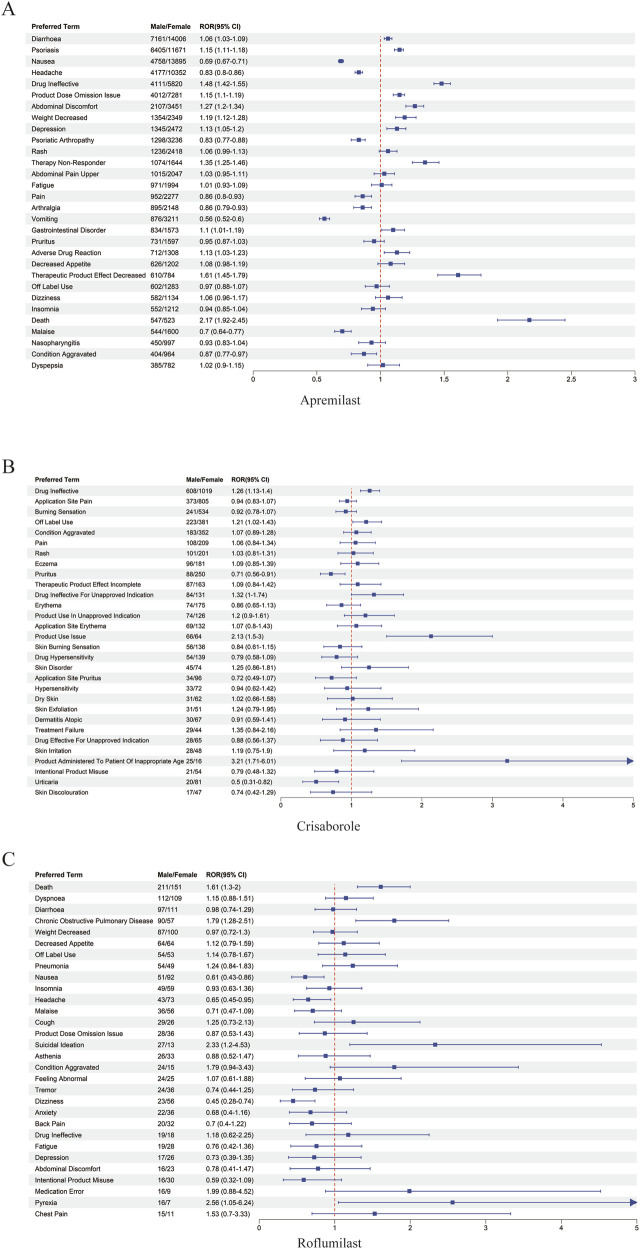
Sex-stratified forest plots for major adverse events associated with the three PDE4 inhibitors. **(A)** Apremilast. **(B)** Crisaborole. **(C)** Roflumilast. Forest plots show male-to-female RORs and 95% CIs for frequently reported preferred terms. Vertical dashed line marks ROR = 1 (no sex difference).

When sex-stratified disproportionality signals were examined across PT levels using $ROR_{ratio}$ and Z-tests, clear, non-random patterns emerged (Figures 2, 3). Rather than isolated outliers, the signals displayed structured gradients that aligned tightly with each agent’s pharmacologic properties and patient population. Across the class, three consistent themes were observed:

#### 3.2.1 Magnitude gradient of sex influence across the class

Apremilast demonstrated a mixed but distinctly female-skewed tolerability profile, with higher female RORs for nausea, diarrhoea and headache. In contrast, Roflumilast showed the strongest male-biased AE elevation, with substantially higher male risks for death, psychiatric symptoms, respiratory exacerbations, metabolic changes, and gastrointestinal AEs. Crisaborole displayed minimal sex-differential amplification, consistent with negligible systemic absorption.

#### 3.2.2 Bidirectional sex imbalance within apremilast

A “dual-direction” structure was evident: males showed elevated RORs for severe or systemic events (death, COPD-like symptoms, suicidal ideation), while females predominated in mucosal, autonomic, and neurologic tolerability events. This pattern reinforces that sex differences reflect mechanistic divergence rather than reporting noise.

#### 3.2.3 Consistent male-dominant amplification with roflumilast

Male users exhibited markedly higher RORs across psychiatric, metabolic, respiratory, and systemic PT categories, forming the steepest sex gradient among the three agents. Even within the same PT category, male RORs frequently exceeded female estimates by large margins, reflecting heightened susceptibility.

Collectively, these observations illustrate that patient sex is associated with significant variations in drug-specific AE signatures in structured and clinically coherent patterns, rather than acting as a random confounder.

### 3.3 SOC-level clustering and organ-system integration

**FIGURE 4:**
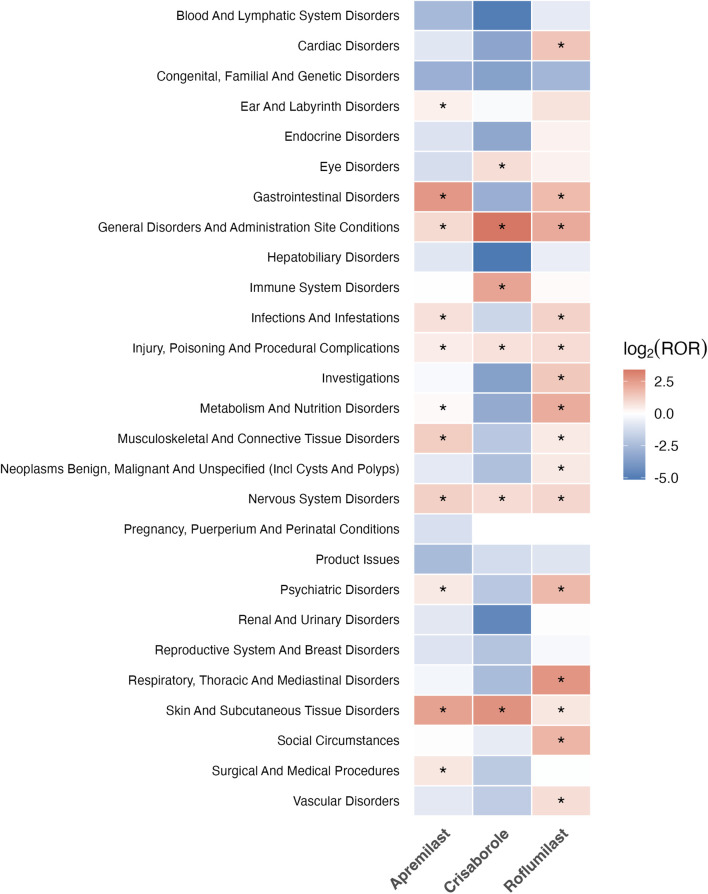
SOC-level disproportionality heatmap for Apremilast, Crisaborole, and Roflumilast. **(A)** Apremilast. **(B)** Crisaborole. **(C)** Roflumilast. Heatmap illustrates system organ class (SOC)–level disproportionality. Colors reflect the magnitude of the log2-transformed reporting odds ratio (ROR): high log2(ROR) values (red) and low log2(ROR) values (blue).

At the organ-system level, each PDE4 inhibitor demonstrated a unique SOC signature (Figure 4). To ensure methodological rigor, SOC-level RORs were calculated based on unique Case IDs rather than PT counts, effectively eliminating potential double-counting bias. Apremilast (N = 127,516) displayed strong enrichment in Gastrointestinal, Nervous System, and General Disorders. Roflumilast (N = 2,142) showed clustering in respiratory, psychiatric, and metabolic SOCs; and Crisaborole was almost exclusively represented within skin and subcutaneous tissue disorders. Importantly, several SOC-level differences were themselves sex-dependent. Females showed stronger neurological and mucosal SOC signals with Apremilast, whereas males showed elevated psychiatric, metabolic, and respiratory SOC signals for Roflumilast. These findings suggest that sex interacts not only with specific symptom types but with broader physiologic and organ-system domains.

### 3.4 Time-to-onset (TTO) divergence and sex-associated temporal patterns

**FIGURE 5:**
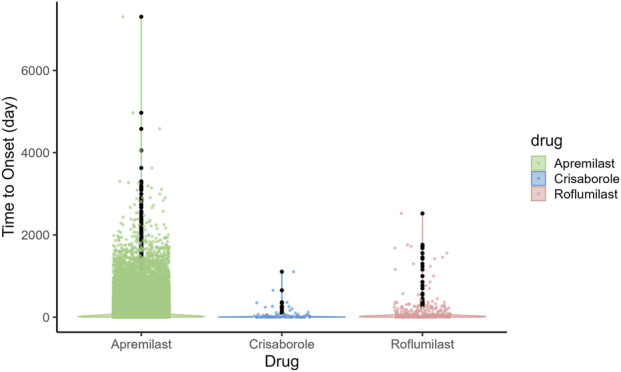
Time-to-onset distributions of adverse events among the three PDE4 inhibitors. **(A)** Apremilast. **(B)** Crisaborole. **(C)** Roflumilast. Scatter–box plots display case-level onset times and median/IQR ranges. The distributions illustrate distinct failure patterns: Crisaborole exhibits early-onset clustering (topical stimulation), while Apremilast and Roflumilast show more diverse and delayed temporal signatures indicative of systemic pharmacological effects.

Marked differences were observed in TTO distributions across the three agents (Figure 5).The median TTO (IQR) was 24 (7–86) days for Apremilast, 4 (1–15) days for Crisaborole, and 42 (14–128) days for Roflumilast. Crisaborole produced rapid-onset events, typically within days, consistent with its topical mechanism. Apremilast demonstrated a broader TTO profile, with gastrointestinal events peaking during the first month of titration. Roflumilast showed a biphasic profile: rapid onset for psychiatric AEs, followed by later-emerging metabolic events. Sex differences were also reflected in onset timing; for instance, females exhibited earlier autonomic and gastrointestinal onset with Apremilast, aligning with the female-biased signal intensity observed in the sex-stratified analysis.

### 3.5 Validation of signals against clinical benchmarks

To validate the reliability of our findings, we compared FAERS-derived signals with FDA product labels and pivotal Phase III trial data (Supplementary Table S6). (Papp et al., 2015; Paller et al., 2016; Gyldenløve et al., 2024) (Administration USFaD, 2011; Administration USFaD, 2014; Administration USFaD, 2016). Common adverse events demonstrated both high RORs and high clinical incidences (e.g., diarrhea for Apremilast), confirming the sensitivity of our model. Crucially, our study also identified signals with high RORs but very low incidence rates in clinical trials (e.g., psychiatric events for Roflumilast and severe mucosal reactions for Apremilast). In pharmacovigilance, these low-incidence/high-ROR signals are of paramount significance as they may represent rare but serious risks that were potentially underpowered in randomized controlled trials, thereby justifying the need for continuous post-marketing surveillance.

### 3.6 Integrated cross-drug, cross-sex AE architecture

Taken together, these multidimensional analyses reveal a coherent class-level architecture in which: (1) pharmacology and route of administration shape baseline risk; (2) patient sex is associated with variations in AE likelihood, reporting severity, and temporal onset; (3) drug identity determines organ-system vulnerability. These findings underscore that PDE4 inhibitors do not share a uniform safety profile but exhibit structured, sex-influenced, drug-specific AE patterns detectable only through large-scale pharmacovigilance interrogation.

## 4 Discussion

This large-scale real-world pharmacovigilance study demonstrates that PDE4 inhibitors share a common anti-inflammatory mechanism shaped by both patient sex and drug-specific pharmacological properties (e.g., systemic absorption and route of administration). Using more than 19 million deduplicated FAERS reports, we identified structured, non-random patterns in sex-differential disproportionality, leading adverse events, organ-system involvement, and time-to-onset patterns. Collectively, these findings highlight that PDE4 inhibitors cannot be viewed as a homogeneous therapeutic class from a safety perspective, and that patient sex acts as a significant biological determinant of adverse event susceptibility within this class (Ibrahim et al., 2021).

A central observation was the gradient of sex influence across the PDE4 inhibitor class. The magnitude of sex-differential reporting followed a consistent pattern, with Roflumilast showing the strongest male-predominant risks, Apremilast demonstrating a female-skewed tolerability profile, and Crisaborole exhibiting minimal sex-specific divergence. This gradient aligns with their differing pharmacokinetic and pharmacodynamic characteristics (Aljohmani and Yildiz, 2025). Roflumilast’s systemic exposure, hepatic metabolism, and catabolic effects may create vulnerability in males, who have distinct inflammatory responses, metabolic profiles, and COPD-related comorbid burden (Klein and Flanagan, 2016) (Kawamatawong, 2021) (Buhl et al., 2012). In contrast, Apremilast’s gastrointestinal and neurologic tolerability profile aligns with known sex differences in autonomic sensitivity, nociception, and immune-mediated reactions, which are heightened among females (Waldman et al., 2007) (Kavanaugh et al., 2019). Crisaborole’s lack of systemic absorption likely explains its flat sex-specific landscape, underscoring how route of administration and systemic exposure govern sex-related risk differences (Dalla and Kokras, 2025) (Silverberg et al., 2024).

The identification of sex-differential clusters of adverse events further suggests that the patterns observed in this study are unlikely to be attributable solely to random noise (Sportiello and Capuano, 2024) (de Vries et al., 2019). Female-predominant neurologic and cardiovascular AEs with Apremilast may be partly influenced by known sex-related differences in autonomic reactivity, β-adrenergic responsiveness, serotonin pathways, and vascular regulation, although these mechanisms have not been specifically validated in the context of PDE4 inhibition (Liccardo et al., 2022) (Ruschil et al., 2021) (Castle and Flanigan, 2024). Conversely, the male-skewed psychiatric and metabolic signals associated with Roflumilast may reflect interactions between the drug’s systemic pharmacology and sex-related differences in dopaminergic modulation, inflammatory signaling, and the greater systemic inflammatory and metabolic burden often observed among male patients with COPD (Cahill, 2006) (Milne et al., 2024). These interpretations remain hypothesis-generating rather than confirmatory, but they are directionally consistent with broader evidence regarding sex-specific susceptibilities to neurologic, cardiovascular, psychiatric, and metabolic adverse drug reactions in real-world populations (Shan et al., 2023). The consistency of these sex-differential signals across multiple disproportionality algorithms further supports their robustness at the signal-detection level, while acknowledging that disproportionality metrics cannot establish causal relationships (Gravel et al., 2024).

At the organ-system level, the three agents demonstrated highly distinctive SOC-level clustering, suggesting that each PDE4 inhibitor engages different physiologic pathways or interacts with distinct comorbidity profiles. Apremilast’s gastrointestinal and neurologic clusters reflect known PDE4-related cAMP elevations affecting emesis pathways and central neurotransmission (Zhao et al., 2013). Roflumilast’s enrichment in respiratory, psychiatric, and metabolic SOCs aligns with its indication in COPD, its known propensity for weight loss and systemic inflammation, and reports of hepatic and metabolic adverse reactions in real-world use (Fabbri and Rabe, 2007) (Gyldenløve et al., 2023). Crisaborole’s concentration in cutaneous SOCs mirrors its topical route of administration and demonstrated low systemic absorption in healthy-volunteer pharmacokinetic studies (Cao et al., 2024). Importantly, several SOC-level differences appear to be sex-dependent, consistent with broader pharmacovigilance evidence showing that sex influences adverse drug reaction profiles across organ systems (Lacroix et al., 2022) (de Vries et al., 2019).

The time-to-onset differences observed among the three PDE4 inhibitors reflect distinct “failure-type” patterns. The sharp, early-onset peak for Crisaborole aligns with an early-failure pattern ( β < 1), characteristic of its topical application. Conversely, the delayed distribution for Apremilast and the biphasic profile of Roflumilast suggest systemic accumulation or adaptation-related mechanisms, potentially linked to their oral route and broader organ-system engagement. For Apremilast, gastrointestinal and neurologic adverse events typically emerge over the first several weeks of treatment in clinical trials, consistent with our finding of a delayed time-to-onset distribution (Paul et al., 2015) (Mease et al., 2023). In contrast, Crisaborole—administered topically with minimal systemic exposure—demonstrates application-site burning or stinging that often occurs shortly after treatment initiation, aligning with the early-onset peak observed in our dataset (Eichenfield et al., 2017) (Jarnagin et al., 2016). For Roflumilast, our data revealed a biphasic pattern in which gastrointestinal and neuropsychiatric events tend to occur early in therapy, whereas metabolic events such as progressive weight loss accumulate over longer durations. This temporal dissociation corresponds with known safety profiles from COPD trials, where early diarrhea and insomnia contrast with longer-term systemic metabolic effects (Au Duong et al., 2005). Together, these time-to-onset signatures offer clinically relevant clues, as early versus delayed patterns are frequently used to help differentiate drug-related from disease-related events in pharmacovigilance assessments.

Collectively, these integrated findings support a conceptual model wherein the safety profile of each PDE4 inhibitor is the combined product of its pharmacologic architecture, route of administration, target population, and sex-dependent biological responses (Younes, 2024). The results emphasize that sex is not an incidental variable but a determinant of real-world drug safety, consistent with pharmacovigilance evidence that females and males exhibit systematically different ADR patterns (Shan et al., 2023). This is in line with growing evidence that females and males differ in immune function, hormonal regulation, metabolism, microbiome ecology, and nociceptive pathways—factors that can directly influence adverse drug reactions (Sharma et al., 2025) (Massey et al., 2020) (Shobeiri et al., 2022). Our study extends this knowledge into the PDE4 inhibitor class and provides class-level clarity using population-scale pharmacovigilance data.

The study also offers practical clinical implications. First, clinicians should anticipate gastrointestinal and neurologic tolerability issues with Apremilast—particularly in subgroups already known to be vulnerable to such events—and may consider slower titration or more proactive counseling (Mease et al., 2020). Second, given Roflumilast’s well-recognized gastrointestinal, psychiatric, and metabolic adverse event profile, together with our observation of male-biased disproportionality signals, closer monitoring of male patients—including early symptom screening for mood changes and weight loss—may help reduce AE burden (Yu et al., 2013) (Cilli et al., 2019). Third, Crisaborole’s predominantly cutaneous and generally mild adverse events, combined with low systemic absorption and reassuring long-term safety across age groups, suggest relatively limited concern regarding sex-specific risk and support its use across diverse patient populations (Paller et al., 2016) (Zane et al., 2016).

Finally, several limitations should be acknowledged. First, FAERS is a spontaneous reporting system subject to underreporting, reporting bias, and a lack of denominator data. Second, the distinct indications and demographic structures—such as older male COPD patients for Roflumilast versus female psoriasis patients for Apremilast—may act as important confounders for the observed sex-biased patterns. Third, while median and interquartile ranges provided a macro-level overview of temporal AE onset, future studies employing Weibull shape parameter ( β ) analysis are warranted to determine the specific failure types (e.g., early-type vs. wear-out type) for individual drug-AE combinations. Despite these constraints, the large sample size and multi-algorithm approach provide a robust signal-detection framework.

## 5 Conclusion

This large-scale real-world evaluation demonstrates that PDE4 inhibitors, despite sharing a common pharmacologic mechanism, exhibit distinct and sex-dependent safety profiles. Using comprehensive FAERS data and multiple complementary disproportionality algorithms, we identified consistent sex-differential AE patterns, drug-specific organ-system clustering, and divergent temporal onset characteristics across Apremilast, Crisaborole, and Roflumilast. These findings reveal that sex is a major modifier of PDE4 inhibitor tolerability and should be incorporated into risk communication and individualized monitoring strategies. Clinically, the results support heightened awareness of female-predominant neurologic and mucosal events with Apremilast, increased vigilance for male-dominant psychiatric and metabolic events with Roflumilast, and confidence in the relatively low sex-related divergence for Crisaborole. Overall, this work provides a class-level framework for understanding PDE4 inhibitor safety and underscores the value of large real-world pharmacovigilance datasets in informing precision medicine.

## Data Availability

Publicly available datasets were analyzed in this study. This data can be found here: https://fis.fda.gov/extensions/FPD-QDE-FAERS/FPD-QDE-FAERS.html.
